# On the Viability of Conspiratorial Beliefs

**DOI:** 10.1371/journal.pone.0147905

**Published:** 2016-01-26

**Authors:** David Robert Grimes

**Affiliations:** University of Oxford, Old Road Campus Research Building, Off Roosevelt Drive, Oxford OX3 7DQ, United Kingdom; University of Waterloo, CANADA

## Abstract

Conspiratorial ideation is the tendency of individuals to believe that events and power relations are secretly manipulated by certain clandestine groups and organisations. Many of these ostensibly explanatory conjectures are non-falsifiable, lacking in evidence or demonstrably false, yet public acceptance remains high. Efforts to convince the general public of the validity of medical and scientific findings can be hampered by such narratives, which can create the impression of doubt or disagreement in areas where the science is well established. Conversely, historical examples of exposed conspiracies do exist and it may be difficult for people to differentiate between reasonable and dubious assertions. In this work, we establish a simple mathematical model for conspiracies involving multiple actors with time, which yields failure probability for any given conspiracy. Parameters for the model are estimated from literature examples of known scandals, and the factors influencing conspiracy success and failure are explored. The model is also used to estimate the likelihood of claims from some commonly-held conspiratorial beliefs; these are namely that the moon-landings were faked, climate-change is a hoax, vaccination is dangerous and that a cure for cancer is being suppressed by vested interests. Simulations of these claims predict that intrinsic failure would be imminent even with the most generous estimates for the secret-keeping ability of active participants—the results of this model suggest that large conspiracies (≥1000 agents) quickly become untenable and prone to failure. The theory presented here might be useful in counteracting the potentially deleterious consequences of bogus and anti-science narratives, and examining the hypothetical conditions under which sustainable conspiracy might be possible.

## Introduction

Conspiratorial beliefs, which attribute events to secret manipulative actions by powerful individuals, are widely held [1] by a broad-cross section of society. Belief in one conspiracy theory is often correlated with belief in others, and some stripe of conspiratorial belief is ubiquitous across diverse social and racial groups [2]. These concepts run the gauntlet from the political to the supernatural, and a single working definition is not easy to obtain. We shall clarify the working definition of conspiracy theory here as being in line the characterisation of Sunstein et al [1] as “*an effort to explain some event or practice by reference to the machinations of powerful people, who attempt to conceal their role (at least until their aims are accomplished)*”.While the modern usage of conspiracy theory is often derogatory (pertaining to an exceptionally paranoid and ill-founded world-view) the definition we will use does not *a priori* dismiss all such theories as inherently false.

However, even with this disclaimer, there are a disconcerting number of conspiracy theories which enjoy popular support and yet are demonstrably nonsensical. This is particularly true of conspiracies over scientific and medical issues where conspiratorial ideation can lead to outright opposition to and rejection of the scientific method [3]. This can be exceptionally detrimental, not only to believers but to society in general; conspiratorial beliefs over medical interventions such as vaccination, for example, can have potentially lethal consequence [4]. Conspiratorial thinking is endemic in anti-vaccination groups, with those advocating the scientific and medical consensus often regarded as agents of some ominous interest group bent on concealing “the truth”. This becomes a defence mechanism to protect beliefs that are incompatible with the evidence, and unsurprisingly perhaps proponents of such views display not only conspiratorial traits but a litany of reasoning flaws, a reliance on anecdote over data and low cognitive complexity in thinking patterns [5].

Similarly, the framing of climate-change as a hoax creates needless uncertainty in public discourse, and increases the risk of damaging inertia instead of corrective action. The dismissal of scientific findings as a hoax also has a political element; a 2011 study found conservative white males in the US were far more likely than other Americans to deny climate change [6]. Similarly, a UK study found that climate-change denialism was more common among politically conservative individuals with traditional values [7]. The public acceptance of climate-change conspiracy transcends the typical wide-ranging domain of conspiratorial belief; a 2013 investigation by Lewandowsky *et al* [8] found that while subjects who subscribed to conspiracist thought tended to reject all scientific propositions they encountered, those with strong traits of conservatism or pronounced free-market world views only tended towards rejecting scientific findings with regulatory implications at odds with their ideological position.

Challenging dubious anti-science assertions is an important element for constructive social debate, and there is some evidence that challenging such narratives can be successful. Belief in the moon-landing hoax is highly associated with acceptance of other conspiracy theories, but there is some evidence that when presented with scientific evidence critical of this narrative that a significant decrease in support for that theory ensues [9]. Previous investigation has also shown that improved communication of knowledge of the scientific consensus can also overcome some conspiratorial thinking on issues as diverse as the link between HIV and AIDs to acceptance of climate-change [10].

Of course, it is worthwhile to take a considered Devil’s advocate approach—there are numerous historical examples of exposed conspiracies and scandals, from Watergate to the recent revelations on the sheer scale of spying on the online activity of citizens by their own governments. It would be unfair then to simply dismiss all allegation of conspiracy as paranoid where in some instances it is demonstrably not so. There is also merit to charges that vested interests can distort and confuse public perception—in the case of climate-change, for example, conservative demagogues have succeeded in casting a perception of doubt on robust science in public discussion [8, 11–14]. Evidently an approach which dismisses these very real concerns out of hand and without due consideration is not good enough, and there must be a clear rationale for clarifying the outlandish from the reasonable.

Something currently lacking that might be useful is a method for ascertaining the likelihood that a conspiracy is viable, and the factors that influence this. The benefits of this would be two-fold; firstly, it would allow one to gauge whether a particular narrative was likely and what scale it would have to operate at. Secondly, and perhaps more usefully, it would help counteract potentially damaging anti-science beliefs by giving an estimate of viability for a conspiracy over time. The parameters for this model are taken from literature accounts of exposed conspiracies and scandals, and used to analyse several commonly held conspiracy theories, and examine the theoretical bounds for the magnitude and time-frame of any posited conspiracy theory.

### 0.1 Anti-Science conspiracy narratives—A brief overview

Conspiracy theories which posit some nefarious underhanded action by scientists are ubiquitous. In these work, we shall restrict our focus to four prominent beliefs of this genre. These are listed below.

1. **NASA Moon-landing conspiracy**—The successful 1969 Apollo 11 mission first put men on the moon, a seminal achievement in human history. Yet even since that historic day, there has been a persistent fringe belief group that strongly believe the moon-landings were faked, mocked up for propaganda purposes. In 2013 it was estimated that 7% of Americans subscribe to this view [15]. Those advocating this conspiracy claim there are inconsistencies in pictures taken on the moon’s surface, despite these claims being comprehensively debunked [16].
2. **Climate change conspiracy**—Climate-change denial has a deep political dimension [7, 8]. Despite the overwhelming strength of evidence supporting the scientific consensus of anthropogenic global warming [17], there are many who reject this consensus. Of these, many claim that climate-change is a hoax staged by scientists and environmentalists [18–20], ostensibly to yield research income. Such beliefs are utterly negated by the sheer wealth of evidence against such a proposition, but remain popular due to an often-skewed false balance present in partisan media [20, 21], resulting in public confusion and inertia.
3. **Vaccination conspiracy**—Conspiratorial beliefs about vaccination are endemic in the anti-vaccination movement [18, 22]. It is estimated that roughly 20% of Americans hold the long de-bunked notion that there is a link between autism and the MMR vaccine [15], a belief which has reduced uptake of important vaccinations [22] in several countries. Anti-vaccination beliefs and scare-mongering are also endemic in the internet age, with vaccine critical websites asserting dubious information [23, 24]. Ill-founded beliefs over vaccination have been darkly successful in stirring panic and reducing vaccine uptake, which has led to damaging resurgence in diseases such as measles [4].
4. **Cancer cure conspiracy**—The belief that a cure for cancer is being withheld by vested interests is a long-standing one [25]. It is often used as a universal deus ex machina for those pushing an alternative alleged cure, and assertion of the conspiracy theory functions as an explanatory device to explain the complete paucity of clinical evidence for such claims [26]. Such claims can be detrimental to patients, some of whom abandon conventional treatment for the lofty but ill-founded promises of alternative medicine [27].

## Methods

### 1.1 Model derivation

We initially assume that for a given conspiracy, conspirators are in general dedicated for the most part to the concealment of their activity. We further assume that a leak of information from any conspirator is sufficient to expose the conspiracy and render it redundant—such leaks might be intentional (in the form of whistle-blowing or defection) or accidental (mistaken release of information). We concern ourselves only with potential intrinsic exposure of the conspiracy and do not consider for now the possibility that external agents may reveal the operation. Thus, it follows that the act of a conspiracy being exposed is a relatively rare and independent event. We can then apply Poisson statistics, and express the probability of at least one leak sufficient to lead to failure of the conspiracy as

$$L=1-e^{-t\phi }$$ (1)

where *ϕ* is the mean number of failures expected per unit time. This is in turn a function of number of conspirators with time *N*(*t*) and *p*, the intrinsic probability of failure per person per year. Then we may specify *ϕ* by

$$\phi =1-{\left(1-p\right)}^{N(t)}$$ (2)

and writing *ψ* = 1 − *p* for brevity, the probability of conspiracy failure can be re-written as a function of time, given by

$$L\left(t,N\left(t\right)\right)=1-e^{-t\left(1-\psi^{N(t)}\right)}$$ (3)

There are several possibilities for the parameter *N*(*t*), the number of conspirators—the appropriate selection will depend on the type of conspiracy involved. If a conspiracy requires constant upkeep then the number required to sustain the fiction is approximately constant with time. This pertains to situations where some active input in either covering up an event or maintaining a deception is vital. In such a case, the number involved takes a simple form of

$$N\left(t\right)=N_{o}$$ (4)

where *N*_*o*_ is the initial number of conspirators. If instead the conspiracy is a single event after which no new conspirators are required then over time those involved will die off, reducing probability of exposure. If this is the case, a Gompertzian survival function can be employed for the function *N*(*t*). If the average age of the conspirators at the moment of the event is *t*_*e*_, then

$$N\left(t\right)=N_{o}e^{\frac{\alpha }{\beta }\left(1-e^{\beta (t+t_{e})}\right)}$$ (5)

where *N*_*o*_ is the initial number of involved individuals, and *α* and *β* are function constants for the Gompertzian curve. For humans, we can use *α* = 10^−4^ and *β* = 0.085 [28] to approximate human mortality. Finally, if conspirators are rapidly removed due to internal friction or otherwise (an action itself which is arguably a meta-conspiratorial event), there may be circumstances where we can model *N*(*t*) as an exponential decay. If members are removed rapidly with only half remaining after a period *t*_2_, then the decay constant is $\lambda =\frac{\text{ln }2}{t_{2}}$ and the number of conspirators at a given time is

$$N\left(t\right)=N_{o}e^{-\lambda t}.$$ (6)

**Fig 1:**
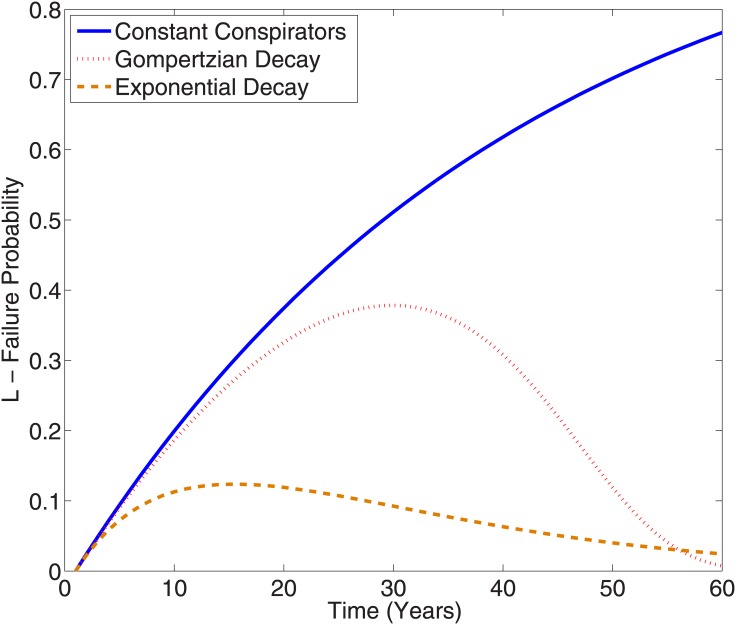
Projected failure probability L for a conspiracy of 5000 initial conspirators and p = 5 × 10^−6^ with different population assumptions. The blue sold line depicts L over time with a constant level of conspirators being maintained. The red dotted line shows a single event with Gompertzian decay of the conspiring population, assuming an average initial age of 40 years old and the dashed orange line shows an exponential decay with number of conspirators being halved every 10 years. In the first case, the likelihood of conspiracy failure always increases with time. In the Gompertzian case, the chances of failure initially increase towards a maximum (L = 0.38 after 29 years in this example), but the death of conspirators with time acts to decrease probability of failure after this. Finally, if conspirators are removed extrinsically, then the curve hits a maximum (L = 0.12 after 14 years) before decaying to lower likelihoods as less conspirators exist to betray confidence.

It is important to note that Eq 6 pivots on the assumption that rapid removal of conspirators doesn’t change the per conspirator probability of exposure—this assumption may not hold in practice and is refined in the discussion section. From Eq 3 it is clear that increasing *N*(*t*) will always act to increase *L*(*t*) no matter what form is chosen for conspirator density. The failure rate with time is slightly more complicated; for the constant case given in Eq 4, *L* will increase monotonically with time. If instead non-constant forms are used, such as those in Eqs 5 and 6, *L* is non-linear with time, as illustrated in Fig 1. The time at which *L* is a maximum in these cases, *t*_*m*_, is given by solving $\frac{\partial L}{\partial t}=0$, which yields the indentity

$$1-\psi^{N}\left(t_{m}\right)\left(1+t_{m}\log\left(\psi \right)\frac{\partial N}{\partial t}{|}_{t_{m}}\right)=0.$$ (7)

This equation is transcendental and cannot be solved analytically, but can be readily estimated by graphical or numerical techniques. The maximum failure probability is then *L*(*t*_*m*_), given by Eq 3. The form of *N*(*t*) shapes the dynamics of the problem markedly, as shown in Fig 1.

### 1.2 Parameter estimation

To use the model, realistic parameter estimates are required. In particular, the parameter *p*, the probability of an intrinsic leak or failure, is extremely important; if *p* were zero, absolute conspiracy would be maintained, only resolvable by extrinsic analysis. In practice, this is not the case—historical examples show that even in incredibly secretive organizations, there is always some possibility of an accidental or intentional intrinsic leak whether by whistle-blowing or ineptitude. By definition, details of conspiracy are rarely known but we may very conservatively estimate parameters using data from exposed examples where sufficient data on duration and number of conspirators is publicly available. The three examples used here are namely

- The National Security Agency (NSA) PRISM affair—The staggering extent of spying by the NSA and its allies on civilian internet users [29] was exposed by contractor Edward Snowden in 2013. The extent of the eavesdropping was unprecedented, including the tapping of fiber-optic cables, phone calls from allied heads of state and a huge amount of meta-data [30].
- The Tuskegee syphilis experiment—In 1932 the US Public Health Service began an observational study on African-American men who had contracted syphilis in Alabama. The study became unethical in the mid 1940s, when penicillin was shown to effectively cure the ailment and yet was not given to the infected men. Ethical questions about the research were raised in the mid 1960s, and finally exposed by researcher Dr. Peter Buxtun in 1972 [31–33].
- The Federal Bureau of Investigation (FBI) forensic scandal—Dr. Frederic Whitehurst wrote hundreds of letters to his superiors detailing the pseudoscientific nature of many of the FBI forensics tests. The dubious nature of these protocols resulted in a large number of innocent men being detained for decades, several of whom were executed for these crimes or died in prison, before Whitehurst exposed the debacle in 1998. A subsequent report by the FBI and Department of justice found that at least 26 of the 28 dedicated hair analysts gave misleading testimony, prompting an on-going massive re-evaluation of unsafe convictions [34, 35].

With data available from these events, we can estimate values for *p* conservatively. We assume that after duration *t* when conspiracies are uncovered that, their probability of failure stands at *L* ≥ 0.5. A lower-bound for *p* is then given by

$$p>1-\sqrt[{N(t)}]{1-\frac{\ln2}{t}}.$$ (8)

There is considerable and unavoidable ambiguity on some of these estimates, especially on the number of people with full knowledge of the event. In the PRISM case, the figure of 30,000 comes from total NSA staff. In reality, the proportion of those employed would would have knowledge of this program would likely be a lot less but we take the upper bound figure to minimize the estimate of *p*. Given the short time-frame involved, we further assume the number of conspirators stayed approximately constant over the duration before the event was exposed. The situation is even more complicated regarding the Tuskegee experiment. This originally fell under the remit of the venereal diseases division of the United States Public Health Service (USPHS) in the early 1930s, before this department was restructured in later years. Historical employment levels for the USPHS are not readily available, so the estimation of 6700 is taken from data for current officer staff levels of the entire USPHS. This likely over-estimates the number involved substantially, which historically would have chiefly concerned only the much smaller venereal disease division. The FBI forensics scandal is also difficult to quantify; while 28 agents were specifically involved with the microscopic hair analysis debacle [39], Dr Whitehurst’s whistle-blowing exposed much wider scale problems affecting the entire forensics department. Accordingly, we have used the modern estimate of FBI forensic staff both scientific and agency. Taking a larger value for *N* tends to over-estimate the ability of a mass of conspirators to retain a secret, yet it allows us to set an extreme lower bound for *p*, the failure odds per unit time per conspirator. This essentially yields a “best-case” scenario for the conspirators.

In addition to this, the life-time of the conspiracy is not always clear—in the NSA case, estimates span only a narrow range, between 5 and 6 years [29]. The Tuskegee experiment is more ambigious; the original experiment commenced in the 1930s but did not become unethical until the late 1940s, when the decision was made to deny penicilin to the afflicted individuals. There were also ethical questions raised by others before Dr. Peter Buxten, but we use 1972 as our upper-limit as it was his whistle-blowing that focused attention on the long-running abuses. Finally, the FBI forensics time-frame is rather opaque—the FBI forensics laboratory was established in 1932, and naively we could take the conspiracy life-time as 66 years before exposure in 1998, in which case this would push the estimate of *p* down by roughly an order of magnitude to *p* > 2.11 × 10^−5^. Yet this is unrealistic, as the problems with certain aspects of nascent criminology were unlikely to have been known. However, between 1992 and 1997 Dr. Whitehurst penned several hundred letters to this superiors about gaping problems with aspects of the analysis, which were roundly ignored. It follows that the FBI were aware from at least 1992 that their forensic methods were untenable, giving a life-time until exposure of only 6 years. In all cases, we take the largest realistic value of *t* as this pertains to the best-case scenario for a conspiracy.

### 1.3 Experimental method

The model established allows estimation of how certain parameters influence the success or failure chance for any conspiracy. From Table 1, assuming the derived best-case scenario value for the conspirators (*p* = 4.09 × 10^−6^), we can apply the model outlined to several popular and enduring conspiracy theories and ascertain their viability with time. As discussed in the previous section, this estimate is intentionally optimistic for conspirators, and corresponds to a case where the average expected number of fatal leaks for a conspiracy is as low as roughly 4 in a million. In keeping with “best case scenario” estimates for conspiracies, we also neglect the upper figure of *p* = 2.45 × 10^−4^, which is roughly 60 times greater than the minimum projected probability of failure per conspirator per year as outlined in Table 1.

**Table 1:** Known and derived parameters.

**NSA PRISM project**	
Maximum involved	30,000 [36]
Time to exposure	6 years^†^
Estimated *p*	4.09 ×10^−6^
Estimated *ψ*	0.99999591
**Tuskegee syphilis experiment**	
Maximum involved	6,700 [37]
Time to exposure	25 years^⋆^
Estimated *p*	4.20 ×10^−6^
Estimated *ψ*	0.99999580
**FBI forensics scandal**	
Maximum involved	500 [38]
Time to exposure	6 years^°^
Estimated *p*	2.45 ×10^−4^
Estimated *ψ*	0.99975500

^†^Other accounts state 5 years—6 picked for conservative estimate [29].

^⋆^Time calculated from unethical experiment duration—1947 to 1972

^°^Time calculated from duration FBI were aware of evidence issues—1992 to 1998

## Results

**Fig 2:**
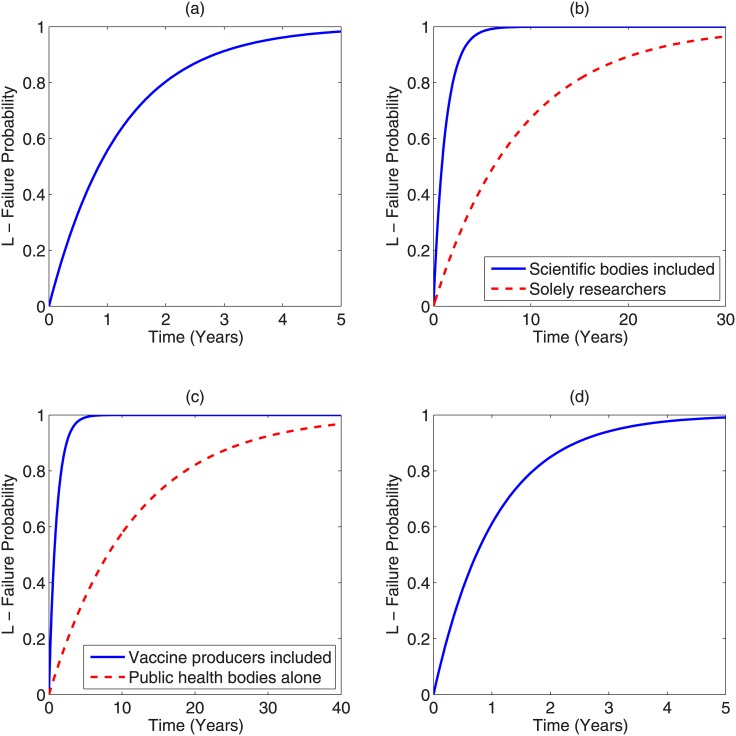
Failure curves for (a) NASA moon-landing hoax—results for both constant population and Gompertzian function are so close as to be non-resolvable visually (b) Climate change hoax—The blue solid line depicts failure probability with time if all scientific bodies endorsing the scientific consensus are involved, the red-dotted line presents the curve if solely active climate researchers were involved (c) Vaccination conspiracy—blue solid line showing failure probability with time for a combination of public health bodies and major drug manufacturers and the red-dotted line depicting case if only public health bodies were conspiring (d) Failure with time for a suppressed cancer cure conspiracy.

Table 2 lists non-exhaustive estimations of the number of conspirators required for the anti-science belief outlined. Critically, the estimates for *N*(*t*) shown here assume all scientists involved would have be aware of an active cover-up, and that a small group of odious actors would be unable to deceive the scientific community for long timescales; the rationale for this assumption is expanded further in the discussion section. In most of these cases, constant up-keep would be required to maintain secrecy, so *N*(*t*) = *N*_*o*_. In the case of the NASA hoax conjecture, it could be argued that the conspiracy was a single-event fiction, and thus the Gompertzian population form in Eq 5 could apply. This is not a very realistic assumption, but is considered here too. The climate-change conspiracy narrative requires some clarification too; those sceptical of the scientific consensus on anthropogenic climate change may take either a “hard” position that climate-change is not occurring or a “soft” position that it may be occurring but isn’t anthropogenic. For this investigation, we’ll define climate change conspiracy as those taking a hard position for simplicity. Results are shown in Fig 2. From this, we can also determine the maximum time-scales before imminent failure under best-possible conditions for these conspiracies, taken as *L* > 0.95. These estimates are given in Table 3.

**Table 2:** Non-exhaustive estimates of minimum numbers needed for conspiracy.

Conspiracy	Employed	Total
**Moon-landing Hoax**		
Peak NASA employment (1965) [40]	411,000	**411,000**
**Climate-change fraud^†^**		
American Geo-Physical Union [41]	62,000	
NASA (Current) [42]	58,000	
American academy for Advancement of Science [43]	120,000	
Royal Society Fellows [44]	16,000	
European Physical Society [45]	120,000	
Published Climate Scientists [46]	≈29,083	
*Total*		≈**405,000**
Vaccination Conspiracy*		
Centre for Disease Control (CDC) [47]	15,000	
World Health Organisation (WHO) [48]	7,000	
*Total*		**22,000**
**Suppressed Cancer cure^⋆^**		
Novartis	65,262	
Pfizer	116,500	
Roche	78,604	
Sanofi	105,000	
Merck and Co.	70,000	
Johnson and Johnson	122,200	
GlaxoSmithKline	99,000	
AstraZeneca	57,500	
*Total*		≈**714,000**

^†^ Estimated from sample memberships of scientific organisations supporting AGW consensus.

* Assuming only major international public health bodies involved in cover-up.

^⋆^ Peak staff numbers for 8 top pharmaceutical companies.

**Table 3:** Maximum time to imminent failure (*L* > 0.95).

Conspiracy	Failure Time
Moon-landing Hoax (Sustained / Constant)	3.68 years
Moon-landing Hoax (Single event / Gompertzian)	3.68 years
Climate-change fraud (Scientists only)	26.77 years
Climate-change fraud—including scientific bodies	3.70 years
Vaccination Conspiracy—CDC/WHO only	34.78 years
Vaccination Conspiracy—including drug companies	3.15 years
Suppressed Cancer cure	3.17 years

## Discussion

The analysis here predicts that even with parameter estimates favourable to conspiratorial leanings that the conspiracies analysed tend rapidly towards collapse. Even if there was a concerted effort, the sheer number of people required for the sheer scale of hypothetical scientific deceptions would inextricably undermine these nascent conspiracies. For a conspiracy of even only a few thousand actors, intrinsic failure would arise within decades. For hundreds of thousands, such failure would be assured within less than half a decade. It’s also important to note that this analysis deals solely with intrinsic failure, or the odds of a conspiracy being exposed intentionally or accidentally by actors involved—extrinsic analysis by non-participants would also increase the odds of detection, rendering such Byzantine cover-ups far more likely to fail. Moreover, the number of actors in this analysis as outlined in Table 2 represent an incredibly conservative estimate. A more comprehensive quantification would undoubtedly drive failure rate up for all considered conspiracy narratives.

This problem appears insurmountable for any large conspiracy; if it requires constant upkeep (*N*(*t*) ≈ *N*_*o*_) then odds of failure approach unity with time. If we assign a detection threshold under which a conspiracy should remain (*μ* = 0.05) in a time-frame, then Table 4 enumerates the maximum number of conspirators possible. Even for a relatively short time of 5 years, the limit is hit with only 2521 agents. To sustain it for more than 10 years, less than 1000 people can be involved even with the generous estimate of *p* = 4.09 × 10^−6^ derived in this work. Even for single-events with Gompertzian population decay, the problem of large conspiracy failure is not adequately circumvented—for such an event, the odds of failure exceed 5% at around 650 participants even with the ideal value of *p* and an average age of participants of 40 years. In this situation however, failure probability eventually falls as the population involved decrease, meaning that the threshold can be considered a maximum probability of detection in this scenario. This probability also rapidly increases with number of conspirators involved, rendering large sustained conspiracies unlikely. Under ideal circumstances, it would only be possible to keep a single conspiratorial event below detection thereshold if the number of actors involved was very small (≪ 1000).

**Table 4:** Maximum number of conspirators to stay below threshold (*μ* ≤ 0.05).

Time frame	Maximum *N*_*o*_
5 years	2531
10 years	1257
15 years	838
20 years	628
25 years	502
30 years	418
40 years	313
50 years	251
100 years	125

**Fig 3:**
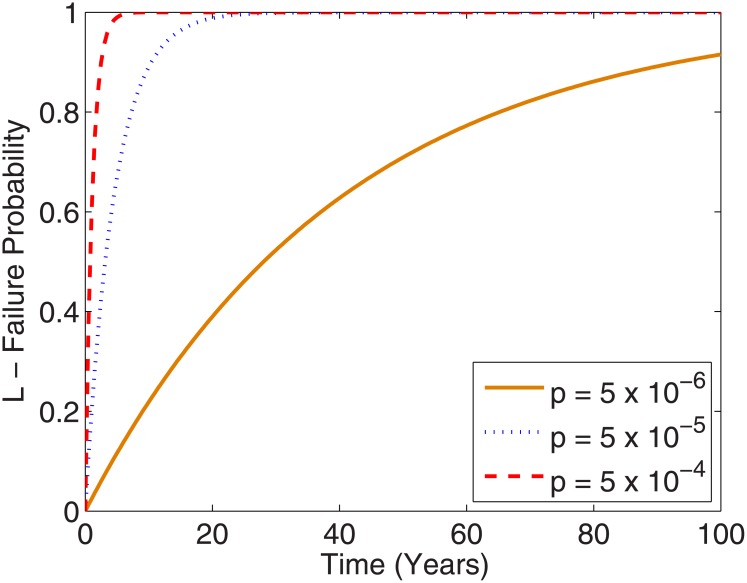
Failure curves for a conspiracy of N_o_ = 5000 as p changes by two orders of magnitude.

As outlined in the section on parameter estimation, estimates used here were deliberately selected to be maximally conducive to conspirators; the lowest values for *p* obtained were used for estimates, but the highest value was roughly two orders of magnitude above this. If this estimate is instead used, it would have a very stark effect, hugely decreasing time-frame to detection as depicted in Fig 3. Given the lack of clarity in getting precise numbers and time-frames, there is inherent uncertainty in this work on the estimated parameters and better estimates would allow better quantification of *p*. There is also an open question of whether using exposed conspiracies to estimate parameters might itself introduce bias and produce overly high estimates of *p*—this may be the case, but given the highly conservative estimates employed for other parameters, it is more likely that *p* for most conspiracies will be much higher than our estimate, as even relatively small conspiracies (such as Watergate, for example) have historically been rapidly exposed. It is also important to note that *p* will likely vary markedly for different conspiracies, depending on how deeply invested agents are invested in a given conspiracy and the figures here are at best a conservative approximation of typical values. However, even if agents are highly invested in a conspiracy, *p* also includes the odds of an accidental intrinsic exposure. While conspiracies do undoubtedly happen, their continued secrecy is probably more due to keeping the number of agents low than having an intrinsically small per agent per time leak probability.

The number of conspirators *N*_*o*_ is also an important uncertainty that needs to be carefully interpreted; the estimates made in this paper (shown in Table 2) are at best order of magnitude estimates. These have deliberately been picked to be relatively conservative in one many respects; for example, the number involved in a hypothetical vaccine conspiracy is likely a massive underestimate due to the ubiquity of vaccination. The estimates also make the assumption that all agents in the estimate are considered to have knowledge of the conspiracy at hand; if this wasn’t the case, then only those with adequate knowledge of the deception would count towards the number *N*_*o*_. This might potentially be the case for some political or social conspiracies, yet for a hypothetical scientific conspiracy it is probably fair to assume that all agents working with the data would have to be aware of any deception. Were this not the case, fraudulent claims or suspect data would be extrinsically exposed by other scientists upon examination of the data in much the same way that instances of scientific fraud are typically exposed by other members of the scientific community. Thus even if a small devious cohort of rouge scientists falsified data for climate change or attempted to cover-up vaccine information, examination by other scientists would fatally undermine the nascent conspiracy. To circumvent this, the vast majority of scientists in a field would have to mutually conspire—a circumstance the model predicts is exceptionally unlikely to be viable.

**Fig 4:**
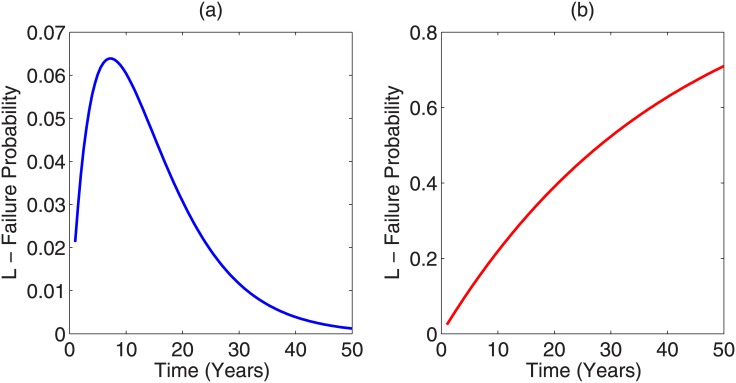
Failure curves for a conspiracy of N_o_ = 5000 over a 50 year period with exponential removal of conspirators with half-life t_2_ of 5 years ($\lambda =\frac{\text{ln }2}{t_{2}}=0.139yr^{-1}$) with (a) assumption of constant p (b) proportional change in probability p(t) = p_o_ e^λt^.

The assumption of Poisson statistics used in this work is justified for discrete events, from cars arriving at a traffic light [49] to radiation induced DNA damage [50] and should hold for exposure of conspiracy events. The model outlined is simple, yet depending on the population function it can yield interesting behaviour. As depicted in Fig 1, the form of *N*(*t*) hugely influences the detection probability of a conspiracy with time. The exponential decay form posited in Eq 6 would in theory yield the lowest probability of detection from a single conspiratorial event, but is likely unrealistic. The reasons for these are twofold—firstly, it implies that conspirators are assassinated or otherwise removed, which itself would be a conspiracy event. But perhaps more relevant is the observation that rapid removal of conspirators would itself likely create panic and disunity amongst invested parties. In this case, *p* would likely become a function of number of conspirators and time. If we assume that the probability of failure increases proportionally to the extinction rate of conspiring parties, then *p*(*t*) = *p*_*o*_ *e*^λ*t*^ then the odds of failure increase dramatically. This behaviour is depicted in Fig 4. For these reasons, the rapid extinction model of population is probably not realistic, even for single-event conspiracy and can be disregarded. In lieu of any available data, we have neglected the potential variation of probability with time in this work, but defining *ψ*(*t*) = 1 − *p*(*t*) (for any suitable *p*(*t*)), we modify Eq 3 to account for this if known by

$$L\left(t,N\left(t\right)\right)=1-e^{-t\left(1-\psi {\left(t\right)}^{N(t)}\right)}.$$ (9)

One of the major motivations is to help counter-act anti-science beliefs from gaining a foothold by quantifying how extraordinarily unlikely it is that a cohesive scientific fraud could take place on such massive scales. This applies not only to the stances examined here, but also to the wild array of popular anti-science beliefs. A sizeable contingent still hold conspiratorial conviction about a range of topics, including Genetically Modified Organisms [51, 52], Water Fluoridation [18, 53, 54], and AIDS Denialism [55, 56] to name but a few prominent examples. It is important to challenge such narratives, as not only are they detrimental to our health and well-being, current research suggests that exposure to conspiratorial beliefs can affect our perception of events to a greater degree than we are aware [57]. Acceptance of such anti-science thinking seems to be correlated with lower resistance to pseudoscientific claims; acceptance of cancer conspiracy claims can drive patients to neglect mainstream medicine in favour of dubious wares by alternative therapists [27]. There is considerable evidence that alternative health practioneers such as homeopaths are far more likely to encourage rejection of vaccination [23, 58, 59] despite their ostensible medical technique being utterly devoid of evidence and completely contradicted by the basic laws of physics [60]. It is unclear whether this relationship is causal or merely correlative.

The theory outlined is useful in predicting the broad patterns expected from a conspiracy event, but does not consider the dynamics, motivations and interactions of individual agents. This interplay might be an avenue for future work, perhaps employing agent based models to account for the various internal frictions and pressures affecting the gross failure rate. The approach outlined here might give some insight into the gross behaviour of conspiracies, but agent based modelling focused on individual actors interacting with certain probabilities might better capture the intricacies of conspiracy and whistle-blowing. Such models could also readily be informed by psychological data, ascribing simulated actors a spectrum of traits, with specific interaction rules to see whether the emergent dynamics affect the success or failure of any secretive event.

While challenging anti-science is important, it is important to note the limitations of this approach. Explaining misconceptions and analysis such as this one might be useful to a reasonable core [9], but this might not be the case if a person is sufficiently convinced of a narrative. Recent work has illustrated that conspiracy theories can spread rapidly online in polarized echo-chambers, which may be deeply invested in a particular narrative and closed off to other sources of information [61]. In a recent Californian study on parents, it was found that countering anti-vaccination misconceptions related to autism was possible with clear explanation, but that for parents resolutely opposed to vaccination attempts to use rational approach further entrenched them in their ill-founded views [62]. The grim reality is that there appears to be a cohort so ideologically invested in a belief that for whom no reasoning will shift, their convictions impervious to the intrusions of reality. In these cases, it is highly unlikely that a simple mathematical demonstration of the untenability of their belief will change their view-point. However, for the less invested such an intervention might indeed prove useful.
