# Correction: On the Viability of Conspiratorial Beliefs

**DOI:** 10.1371/journal.pone.0151003

**Published:** 2016-03-01

**Authors:** David Robert Grimes

In the Methods section, there is an error in the first equation in the subsection titled “1.1 Model derivation.” The equation in the original paper supposes a homogenous Poisson process. This is indeed the case when *ϕ*(*t*) is a constant, which describes the case in the paper when *N*(*t*) = *N*_*o*_ (constant population) but is not justified when *ϕ*(*t*) is a time-varying function [2]. To account for the general case, this equation should read as follows:

$$L=1-e^{-\int_{0}^{t}\phi (t)dt}.$$

In the Methods section, there is an error in the third equation in the subsection title “1/1 Model derivation.” To account for the general case as above, the equation should read as follows:

$$L\left(t,\;N\left(t\right)\right)\;=\;1\;-\;e^{-\;\int_{0}^{t}\left(1\;-\;\psi^{N\;\left(t\right)}\right)\;dt}$$

These corrections do not change the conclusions of the paper, which assumed a constant population of conspirators, but should be kept in mind if one is to apply the model to inhomogeneous populations. When the corrections above are considered, all curves are monotonically increasing with time so equation 7 can be disregarded. Finally, equation 9 with time-varying *p*(*t*) can be described by the corrected equation 2 above.

These corrections do not modify the conclusions of the paper but do impact Figs 1 and 4. Please see the corrected Figs 1 and 4 here.

**Fig 1:**
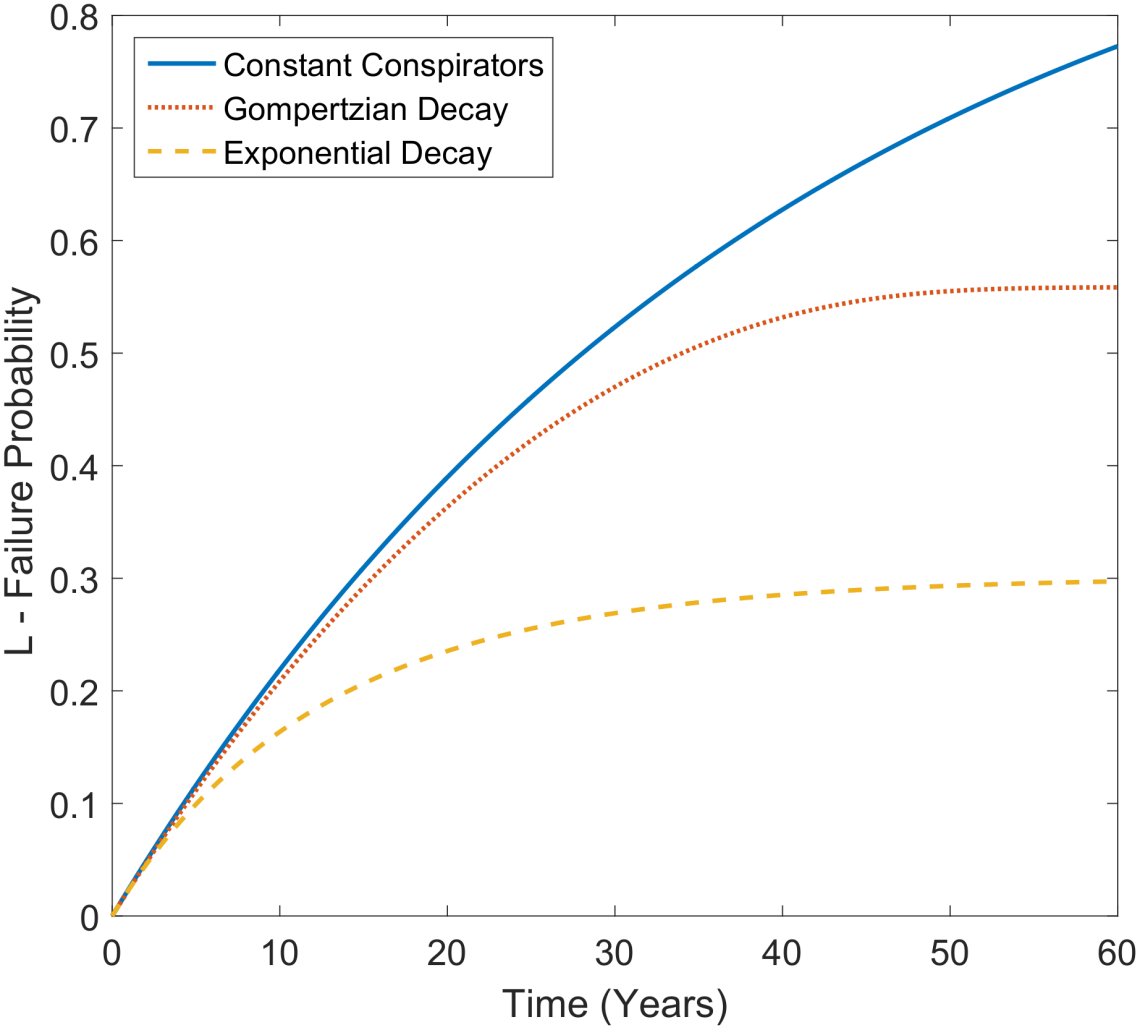
Projected failure probability *L* for a conspiracy of 5000 initial conspirators and *p* = 5×10^−6^ with different population assumptions. The blue sold line depicts *L* over time with a constant level of conspirators being maintained. The red dotted line shows a single event with Gompertzian decay of the conspiring population, assuming an average initial age of 40 years old and the dashed orange line shows an exponential decay with number of conspirators being halved every 10 years.

**Fig 4:**
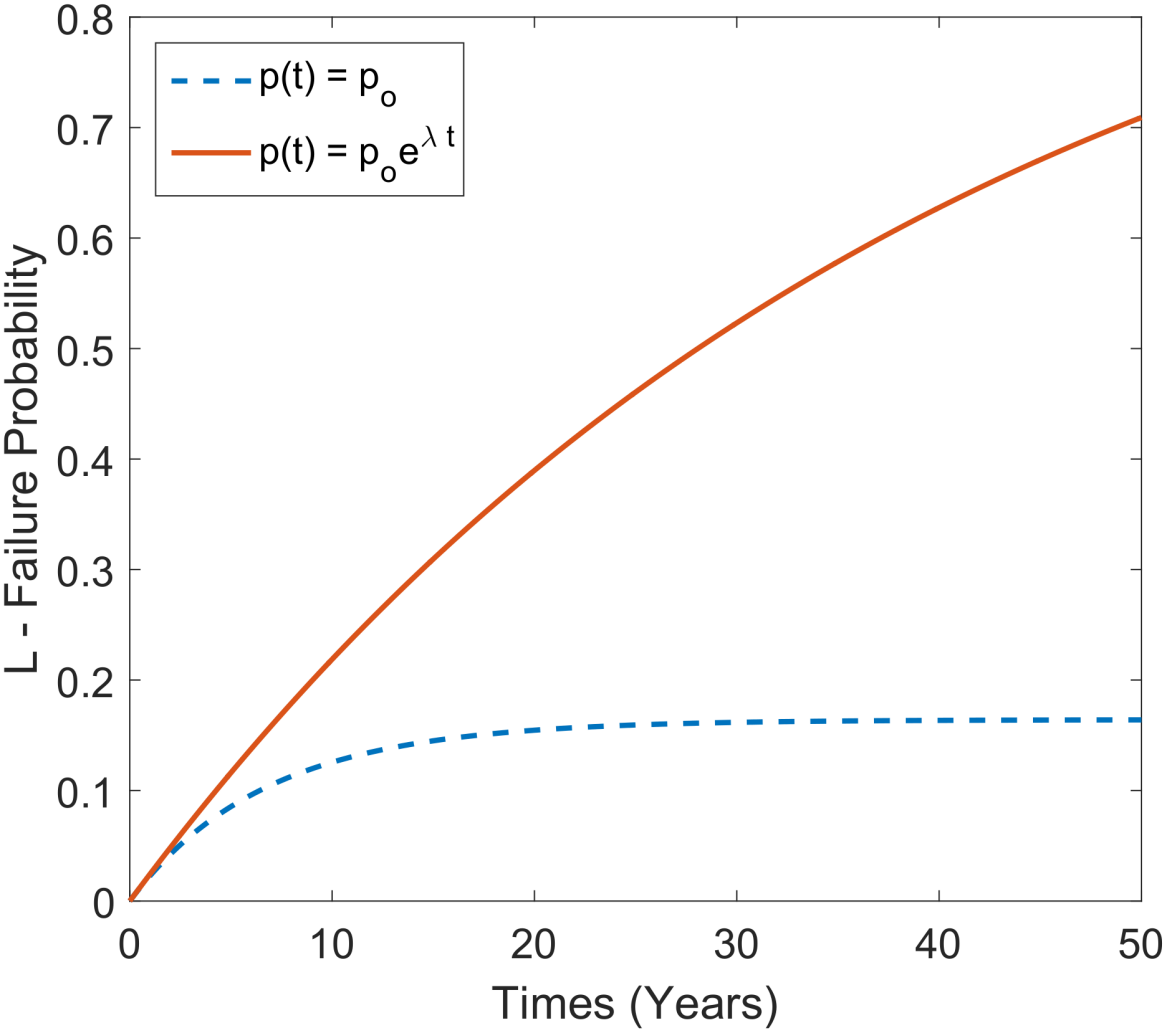
Failure curves for a conspiracy of *N*_*o*_ = 5000 over a 50 year period with exponential removal of conspirators with half-life *t*_2_ of 5 years ($\lambda =\frac{\text{ln}2}{t_{2}}=0.139yr^{-1}$) with assumption of constant p and proportional change in *p* of *p*(*t*) = *p*_*o*_*e*^*λt*^.

There is an error in the fifth sentence of the second paragraph of the Discussion. The correct sentence is: Even for single-events with Gompertzian population decay, the problem of large conspiracy failure is not adequately circumvented—for such an event, the odds of failure exceed 5% within 10 years at around 1328 participants even with the ideal value of *p* and an average age of participants of 40 years.

These corrections to the Discussion do not modify the conclusions of the paper, such as the implausibility of large conspiracies avoiding exposure, as these were all calculated for the homogeneous case with a constant number of conspirators considered. These corrected forms do suggest that the probability of a massive group of conspirators successfully hiding even a single event is even lower than originally outlined.
